# Acute DNA damage activates the tumour suppressor p53 to promote radiation-induced lymphoma

**DOI:** 10.1038/ncomms9477

**Published:** 2015-09-24

**Authors:** Chang-Lung Lee, Katherine D. Castle, Everett J. Moding, Jordan M. Blum, Nerissa Williams, Lixia Luo, Yan Ma, Luke B. Borst, Yongbaek Kim, David G. Kirsch

**Affiliations:** 1 Department of Radiation Oncology, Duke University Medical Center, Durham, North Carolina 27710, USA; 2 Department of Pharmacology and Cancer Biology, Duke University Medical Center, Durham, North Carolina 27710, USA; 3 Department of Population Health and Pathobiology, College of Veterinary Medicine, North Carolina State University, Raleigh, North Carolina 27606, USA; 4 Laboratory of Veterinary Clinical Pathology, College of Veterinary Medicine, Seoul National University, Gwanak-ro, Gwanak-gu, Seoul 151-742, South Korea

## Abstract

Genotoxic cancer therapies, such as chemoradiation, cause haematological toxicity primarily by activating the tumour suppressor p53. While inhibiting p53-mediated cell death during cancer therapy ameliorates haematologic toxicity, whether it also impacts carcinogenesis remains unclear. Here we utilize a mouse model of inducible *p53* short hairpin RNA (shRNA) to show that temporarily blocking p53 during total-body irradiation (TBI) not only ameliorates acute toxicity, but also improves long-term survival by preventing lymphoma development. Using *Kras*^*LA1*^ mice, we show that TBI promotes the expansion of a rare population of thymocytes that express oncogenic *Kras*^*G12D*^. However, blocking p53 during TBI significantly suppresses the expansion of *Kras*^*G12D*^-expressing thymocytes. Mechanistically, bone marrow transplant experiments demonstrate that TBI activates p53 to decrease the ability of bone marrow cells to suppress lymphoma development through a non-cell-autonomous mechanism. Together, our results demonstrate that the p53 response to acute DNA damage promotes the development of radiation-induced lymphoma.

## Abstract (web summary)

p53 can be activated by oncogenic stress to suppress tumourigenesis, but its role in radiation carcinogenesis has not been studied in p53 wild-type mice. Here, Lee *et al*. show that knocking down p53 during total-body irradiation not only reduces acute toxicity, but prevents the formation of radiation-induced lymphoma.

## Graphical abstract

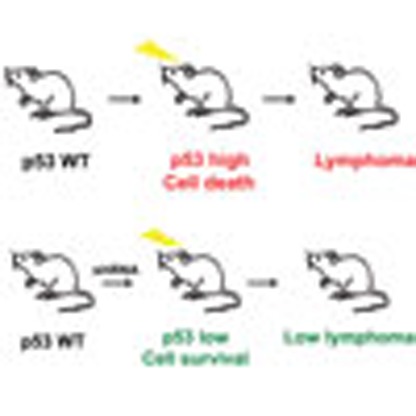

Over 50% of cancer patients receive radiation therapy alone or in combination with chemotherapies during their course of illness^1^. However, the effectiveness of chemoradiation may be limited due to acute normal tissue toxicity. In certain tissues, such as the haematopoietic system, acute toxicity from chemoradiation primarily results from the activation of the intrinsic pathway of apoptosis mediated by the tumour suppressor p53 (refs 2, 3, 4, 5, 6). Thus, blocking p53-mediated apoptosis during genotoxic therapies has been suggested as a promising approach to ameliorate acute toxicity in normal tissues that are wild type (WT) for *p53* without compromising the therapeutic response of *p53* mutant tumours^3^,^7^,^8^.

However, mice with germline deletion of *p53* are susceptible to radiation-induced cancer^9^,^10^. Therefore, one concern with blocking p53-mediated apoptosis during genotoxic therapies is that this approach might exacerbate the development of therapy-related cancer, a major cause of long-term morbidity and mortality for cancer survivors^11^,^12^,^13^. Prior work by Christophorou and colleagues examined the role of p53 in the development of thymic lymphoma after total-body irradiation (TBI) utilizing *p53ER* knock-in mice that were functionally p53 null in the absence of tamoxifen treatment^14^. They showed that treatment with tamoxifen to restore p53 during TBI markedly induced apoptosis, but did not impact lymphoma formation after radiation exposure. In another study, Hinkal *et al*.^15^ used an inducible Cre-loxP model to permanently delete *p53* in somatic cells before, concurrently with, or after TBI. However, these mice showed no difference in the latency of lymphoma formation regardless of when *p53* was deleted. These results demonstrate that the acute p53 response to radiation is dispensable for suppressing the development of lymphomas in mice that have permanently lost *p53*.

In contrast to these elegant mouse models, most cancer patients do not harbour germline *p53* mutations. Instead, they acquire somatic mutations during cell division^16^ or as a consequence of exposure to an environmental mutagen. In this study, to explore the role of p53 in radiation carcinogenesis in a model system with intact *p53*, we use transgenic mice that harbour an inducible *in vivo* shRNA against *p53* (ref. 17) to ask a reciprocal question: what happens to tumour development when p53 is temporarily turned off during irradiation in *p53* WT mice? To our surprise, we find that knockdown of *p53* during TBI not only ameliorates acute haematologic toxicity, but also improves long-term survival of mice by preventing the formation of thymic lymphoma.

## Results

### Blocking p53 during TBI reduces haematological toxicity

**Figure 1:**
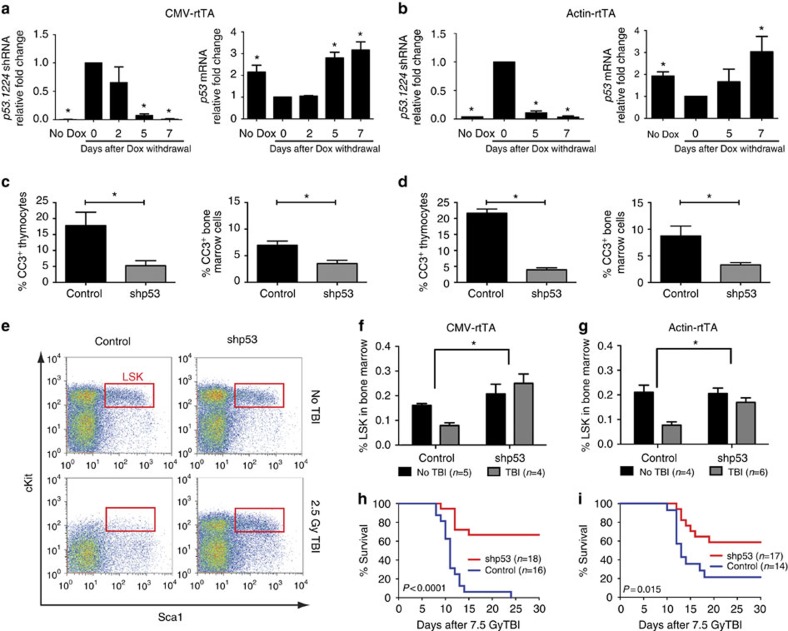
Temporary and reversible induction of p53.1224 shRNA protects mice against acute radiation toxicity of the haematopoietic system. (**a**,**b**) *CMV-rTA; TRE-p53.1224* and *Actin-rtTA; TRE-p53.1224* mice were fed a Dox-containing diet for 10 days and then euthanized at different time points after being switched to a regular diet. Expression of p53.1224 shRNA and p53 mRNA in the bone marrow was assessed by quantitative RT–PCR (*n*=3 mice per group per time point). *P<0.05 for samples compared with 0 days after Dox withdrawal by Student’s *t*-test. Data are presented as mean±s.e.m. (**c**–**i**), *CMV-rtTA; TRE-p53.1224* and *Actin-rtTA; TRE-p53.1224* mice (shp53) as well as their littermates containing either an rtTA or a TRE-p53.1224 allele (control) were treated with Dox for 10 days before irradiation, and were switched to a Dox-free diet immediately after irradiation. (**c**,**d**) The percentage of cleaved caspase-3 positive (CC3^+^) thymocytes and bone marrow cells 4 h after 2.5 Gy TBI (*n*=3 mice per group). *P<0.05 by Student’s *t*-test. Data are presented as mean±s.e.m. (**e**) Representative flow cytometry dot plots of Lineage^−^ Sca1^+^ cKit^+^ (LSK) cells in the bone marrow of shp53 and control mice 24 h after 2.5 Gy TBI or no TBI. cKit and Sca1 were gated from lineage negative cells. (**f**,**g**) The percentage of LSK cells in the bone marrow from shp53 and control mice 24 h after 2.5 Gy TBI or no TBI. *P<0.05 by two-way analysis of variance. Data are presented as mean±s.e.m. (**h**,**i**) Overall survival of shp53 and control mice after exposure to 7.5 Gy TBI, which causes the haematopoietic acute radiation syndrome. *P* value was calculated by log-rank test.

To temporarily and reversibly control p53 expression *in vivo*, we utilized *TRE-p53.1224* transgenic mice in which expression of a miR-30-based p53.1224 shRNA is regulated by a tetracycline-responsive element (TRE)^17^. *TRE-p53.1224* mice were crossed to either cytomegalovirus (*CMV*)-reverse tetracycline-controlled transactivator *(rtTA)*^17^ or β-actin (*Actin*)-*rtTA*^18^ mice to generate compound transgenic mice in which p53.1224 shRNA can be induced ubiquitously by doxycycline (Dox). Treating *CMV-rtTA; TRE-p53.1224* and *Actin-rtTA; TRE-p53.1224* mice with a Dox-containing diet for 10 days induced p53.1224 shRNA that caused a corresponding decrease in p53 mRNA expression in the bone marrow. Withdrawal of Dox decreased expression of p53.1224 shRNA and p53 mRNA was restored within 7 days (Fig. 1a,b). Treatment with Dox for 10 days significantly suppressed radiation-induced apoptosis in thymocytes and bone marrow cells of *CMV-rtTA; TRE-p53.1224* and *Actin-rtTA; TRE-p53.1224* mice (referred to as shp53 mice hereafter) compared with littermates that contain only an rtTA or a TRE-p53.1224 allele (referred to as control mice hereafter; Fig. 1c,d). Moreover, temporary knockdown of p53 during TBI significantly protected Lineage^−^ Sca1^+^ cKit^+^ (LSK) cells, which are enriched for haematopoietic stem/progenitor cells (HSPCs)^19^, against radiation injury (Fig. 1e–g) and improved survival of mice from the haematopoietic acute radiation syndrome^20^ (Fig. 1h,i). Together, these results indicate that temporarily blocking p53 during TBI by shRNA ameliorates acute radiation toxicity of the haematopoietic system.

### Blocking p53 during TBI suppresses lymphoma development

**Figure 2:**
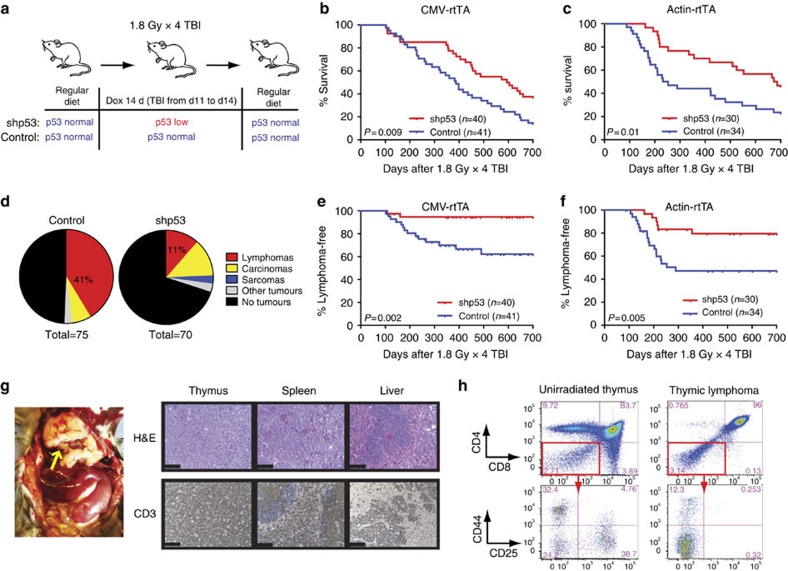
Temporary knockdown of p53 during TBI prevents the development of thymic lymphoma in p53 WT mice. (**a**) *CMV-rtTA; TRE-p53.1224* and *Actin-rtTA; TRE-p53.1224* (shp53) mice as well as their littermates that contain only a rtTA or a TRE-p53.1224 allele (control) were fed a Dox-containing diet for 10 days. Mice were maintained on Dox for an additional 4 days, they were exposed to 4 daily fractions of 1.8 Gy TBI. All mice were switched to a regular diet after the last dose of radiation. (**b**,**c**) Overall survival of mice after TBI. Three control mice from the CMV-rtTA cohort developed the acute radiation syndrome within 30 days after TBI and were excluded from the analysis. *P* value was calculated by log-rank test. (**d**) Spectra of tumours developed in mice after TBI. (**e**,**f**) Lymphoma-free survival of mice after TBI. *P* value was calculated by log-rank test. (**g**) Representative image of a mouse that developed thymic lymphoma (yellow arrow). Representative tissue sections of lymphomas that developed in the thymus, spleen and liver stained with haematoxylin and eosin or anti-mouse CD3 antibody. Scale bar, 100 μm. (**h**) Representative flow cytometry plots of unirradiated thymocytes and lymphoma cells.

To evaluate how blocking p53-mediated radiation toxicity impacts radiation-induced carcinogenesis, *CMV-rtTA; TRE-p53.1224* and *Actin-rtTA; TRE-p53.1224* mice as well as their littermate controls were fed a Dox-containing diet for 14 days. On the final 4 days, the mice received daily fractions of 1.8 Gy TBI (Fig. 2a), which effectively induces haematological cancers in p53 WT mice^21^. Unexpectedly, temporary knockdown of p53 during 1.8 Gy × 4 TBI significantly improved the overall survival of the mice (Fig. 2b,c), which was primarily due to a marked decrease in the percentage of mice that developed lymphomas (Fig. 2d–f). Radiation-induced lymphomas primarily developed in the thymus and, in some mice, disseminated to other organs including the spleen and the liver (Fig. 2g). Lymphoma cells were positive for the T-cell marker CD3 (Fig. 2g), but aberrantly expressed CD4 and CD8 compared with thymocytes from unirradiated mice (Fig. 2h). Molecular characterization revealed that radiation-induced lymphomas overexpressed oncogenic drivers frequently altered in human and mouse T-cell acute lymphoblastic leukaemia^22^,^23^, including the intracellular domain of Notch (ICN), c-Myc and *Lmo2* (Supplementary Fig. 1a,b). In addition, radiation-induced lymphomas maintained intact p53 transcriptional activities following DNA damage (Supplementary Fig. 1c,d) and did not show elevated levels of p19^Arf^ (Supplementary Fig. 1e), indicating that these lymphomas retained functional p53 (ref. 24). As a control, we repeated the TBI experiment in *Actin-rtTA; TRE-p53.1224* mice and littermate controls in the absence of Dox, and observed that the presence of one or two transgenic alleles in the absence of Dox does not influence tumour susceptibility after TBI (Supplementary Fig. 2). Collectively, these data demonstrate that blocking p53 during the acute DNA damage response prevents radiation-induced lymphoma in p53 WT mice.

### TBI activates p53 to expand thymocytes with aberrant TCRβ

**Figure 3:**
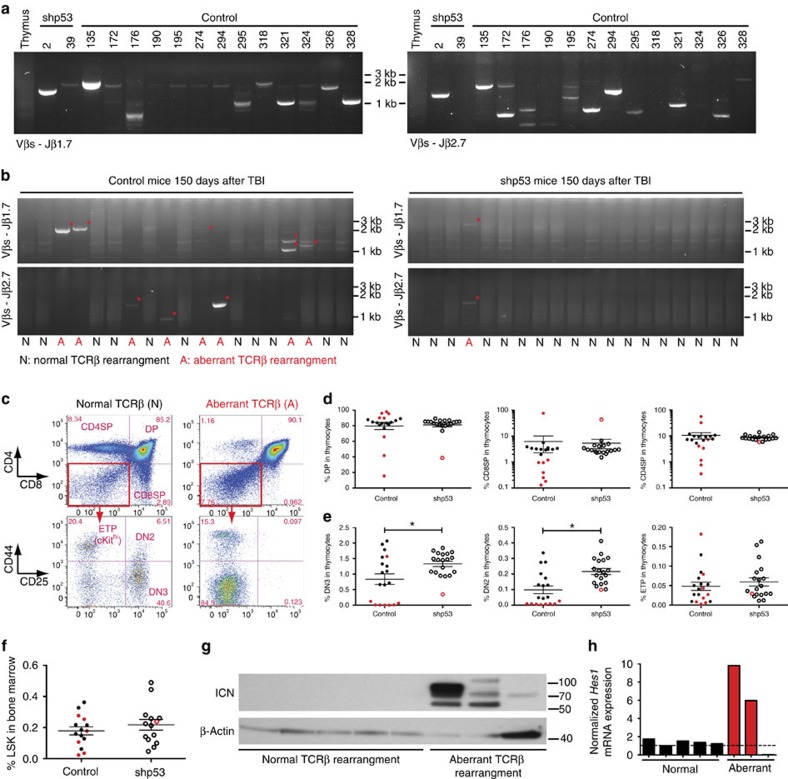
Temporarily blocking p53 during TBI suppresses aberrant expansion of premalignant thymocytes. (**a**) PCR that assesses V–J recombination of the TCRβ locus of radiation-induced lymphomas that developed in either shp53 or control mice. The thymus from an unirradiated mouse was used as a control. (**b**) PCR assessing V–J recombination of the TCRβ locus of thymocytes from control and shp53 mice 150 days after 1.8 Gy × 4 TBI. Red asterisks indicate aberrant PCR bands. (**c**) Representative flow cytometry dot plots of thymocytes having normal (N) and aberrant (A) TCRβ rearrangement. Cells at different stages of thymocyte development were gated based on the expression of surface markers. CD4 single positive (CD4SP); CD8 single positive (CD8SP); DP: CD4^+^CD8^+^; DN3 : CD44^−^CD25^+^CD4^−^CD8^−^; DN2: CD44^+^CD25^+^CD4^−^CD8^−^; ETP: cKit^hi^CD44^+^CD25^−^CD4^−^CD8^−^. (**d**–**f**) The percentage of cells at various stages of thymocyte development and the percentage of LSK cells in the bone marrow from control and shp53 mice 150 days after 1.8 Gy × 4 TBI. Black and red dots represent mice harbouring normal and aberrant TCRβ rearrangement, respectively. *P<0.05 by Students *t*-test. Data are presented as mean±s.e.m. (**g**) Expression of the ICN protein and (**h**) *Hes1* mRNA in thymocytes having normal (N) and aberrant (A) TCRβ rearrangement.

Assessment of the T-cell antigen receptor β (TCRβ) locus showed that most radiation-induced lymphomas contained only one or two dominant rearrangements (Fig. 3a), suggesting that lymphomas developed from the expansion of one or a small number of cells in the irradiated thymus. To study how blocking p53 during TBI affects radiation-induced clonal expansion in the thymus at a premalignant stage, we assessed the TCRβ locus of thymocytes from shp53 and control mice 150 days after 1.8 Gy × 4 TBI. Aberrant rearrangement of the TCRβ locus was detected in 8 out of 19 control mice 150 days after 1.8 Gy × 4 TBI, but in only 1 out of 19 shp53 littermates (*P*<0.05 by Fisher’s exact test; Fig. 3b). In mice harbouring aberrant TCRβ rearrangement (*n*=9), flow cytometry analysis revealed expansion of CD4^+^CD8^+^ double positive cells (*n*=5), CD4 single positive (CD4SP) cells (*n*=2) or CD8 single positive (CD8SP) cells (*n*=2; Fig. 3c,d). Remarkably, additional flow cytometry analysis of the thymocytes also showed that irradiated control mice had a significant decrease in cells at the double negative 3 (DN3) and double negative 2 (DN2) stages of thymocyte development compared with thymocytes from irradiated shp53 mice (Fig. 3e). However, at 150 days after TBI the thymus from irradiated control and shp53 mice had a similar percentage of earliest thymic precursor cells (ETPs), which are thymocyte progenitors from the bone marrow^19^ (Fig. 3e). Moreover, at 150 days after TBI these mice had a similar percentage of LSK cells in the bone marrow (Fig. 3f). Compared with normal thymocytes, thymocytes with aberrant TCRβ rearrangement expressed elevated levels of ICN with increased expression of *Hes1*, a Notch target gene^22^ (Fig. 3g,h). Taken together, these data suggest that temporarily blocking p53 during TBI suppresses the expansion of thymocytes that have acquired oncogenic potential.

### TBI activates p53 to expand Kras^G12D^-expressing thymocytes

**Figure 4:**
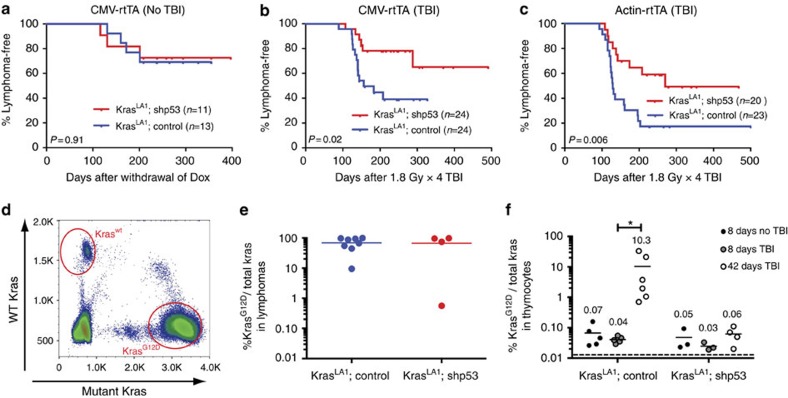
Temporary knockdown of p53 during TBI suppresses expansion of thymocytes expressing oncogenic *Kras*^*G12D*^. (**a**) *Kras*^*LA1*^*; CMV-rtTA; TRE-p53.1224* (Kras^LA1^; shp53) and littermates harbouring a Kras^LA1^ allele alone or in combination with either a rtTA or a TRE-p53.1224 allele (Kras^LA1^; control) were fed a Dox-containing diet for 14 days and then were switched to a regular diet without radiation treatment (no TBI). *P* value was calculated by log-rank test. (**b**,**c**) *Kras*^*LA1*^*; CMV-rtTA; TRE-p53.1224* and *Kras*^*LA1*^*; Actin-rtTA; TRE-p53.1224* (Kras^LA1^; shp53) mice as well as their littermate controls (Kras^LA1^; control) were temporarily treated with Dox during 1.8 Gy × 4 TBI according to the protocol illustrated in Fig. 1a. Two control mice from the CMV-rtTA cohort and one control mouse from the Actin-rtTA cohort developed the acute radiation syndrome within 30 days after TBI and were excluded from the analysis. *P* value was calculated by log-rank test. (**d**) Representative data of droplet digital PCR assessing the frequency of the WT *Kras* allele and the recombined *Kras*^*G12D*^ allele in a thymic lymphoma. (**e**) The percentage of the recombined *Kras*^*G12D*^ allele in thymic lymphomas from irradiated Kras^LA1^; control and Kras^LA1^; shp53 mice. Data are presented as mean. (**f**) The percentage of the recombined *Kras*^*G12D*^ allele in thymocytes from *Kras*^*LA1*^*; CMV-rtTA; TRE-p53.1224* (Kras^LA1^; shp53) mice and littermate controls (Kras^LA1^; control) 8 and 42 days after TBI as well as 8 days after Dox withdrawal without irradiation (8 days no TBI). *P<0.05 by Student’s *t*-test. The numbers represent the mean of each experimental group. The dashed line indicates the background of the assay for detecting the recombined *Kras*^*G12D*^ allele.

We hypothesized that blocking p53 during TBI could inhibit the expansion of premalignant thymocytes by killing tumour-initiating cells in a cell-autonomous manner as a consequence of p53 restoration^14^ and/or by preventing the clonal expansion of tumour-initiating cells through a non-cell-autonomous mechanism^25^. To test this hypothesis in a model with a defined initiating oncogene, we studied radiation-induced carcinogenesis using *Kras*^*LA1*^ mice^26^, in which spontaneous thymic lymphomas are initiated by thymocytes in which the *Kras*^*LA1*^ allele has undergone intrachromosomal recombination to express oncogenic *Kras*^*G12D*^. In the absence of radiation, knockdown of p53 by Dox treatment for 14 days did not influence the penetrance or latency of thymic lymphoma development (Fig. 4a). However, temporary knockdown of p53 during 1.8 Gy × 4 TBI significantly suppressed lymphoma formation in both *Kras*^*LA1*^*; CMV-rtTA; TRE-p53.1224* and *Kras*^*LA1*^*; Actin-rtTA; TRE-p53.1224* mice (referred to as Kras^LA1^; shp53 mice hereafter) compared with littermate controls (Fig. 4b,c). Quantification of the recombined *Kras*^*G12D*^ mutant allele on a single-molecule basis by droplet digital PCR showed that most radiation-induced lymphomas that developed in *Kras*^*LA1*^ mice were initiated by cells harbouring oncogenic *Kras*^*G12D*^ (Fig. 4d,e). To examine the persistence of the oncogenic *Kras*^*G12D*^ mutation in thymocytes after p53 restoration in the presence and absence of irradiation, we assessed the percentage of the recombined *Kras*^*G12D*^ allele in Kras^LA1^; shp53 mice and littermate controls at various time points after TBI. At day 8 after TBI, the percentage of the recombined *Kras*^*G12D*^ allele in thymocytes of Kras^LA1^; control mice was similar to thymocytes of unirradiated Kras^LA1^; control mice (mean=0.04 and 0.07% for mice with and without irradiation, respectively; Fig. 4f), suggesting that radiation did not increase the pool of lymphoma-initiating cells at this time. However, the percentage of the recombined *Kras*^*G12D*^ allele was markedly increased in thymocytes from Kras^LA1^; control mice 42 days after TBI (mean=0.04 and 10.3% for mice 8 and 42 days after TBI, respectively; Fig. 4f), indicating that radiation promotes the expansion of cells expressing *Kras*^*G12D*^. Remarkably, in Kras^LA1^; shp53 mice, the percentage of the recombined *Kras*^*G12D*^ allele in thymocytes was not significantly different between mice with and without irradiation at day 8 (mean=0.03 and 0.05% for mice with and without irradiation, respectively; Fig. 4f) as well as between irradiated mice at day 8 and day 42 (mean=0.03 and 0.06% for mice 8 and 42 days after TBI, respectively; Fig. 4f). These results do not exclude the possibility that restoration of p53 after TBI may eliminate some lymphoma-initiating cells with a mutation that requires p53 knockdown for survival^14^,^27^,^28^, but the droplet digital PCR results demonstrate that thymocytes with oncogenic *Kras*^*G12D*^ remain after p53 restoration and fail to undergo clonal expansion. Therefore, these data indicate that blocking the p53 response to acute DNA damage suppresses the progression of radiation-induced lymphomas in *Kras*^*LA1*^ mice.

Because *Kras*^*LA1*^ mice develop lung cancers with 100% penetrance^26^, in mice that did not develop lymphoma, we also compared the overall survival and aggressiveness of lung cancer in irradiated Kras^LA1^; shp53 (*n*=29) and Kras^LA1^; control (*n*=14) mice. We found that temporary knockdown of p53 did not significantly influence the survival of mice that succumbed to lung cancers (Supplementary Fig. 3a). In addition, the area of the lung with disease (Supplementary Fig. 3b), the percentage of lung tumours positive for phospho-p44/42 MAPK (pErk; Supplementary Fig. 3c), and the grade of the lung tumours (Supplementary Fig. 3d,e) were not significantly different in shp53 and control mice. Therefore, blocking p53 during TBI impairs radiation-induced lymphoma formation without exacerbating lung tumour development in *Kras*^*LA1*^ mice.

### Lymphocyte death and lymphomagenesis of shp53 mice

Recent studies showed that *PUMA* knockout mice, which have defects in p53-dependent apoptosis, are resistant to radiation-induced thymic lymphoma^29^,^30^. Notably, *PUMA* knockout mice were resensitized to radiation-induced lymphoma when irradiation was combined with dexamethasone treatment, which causes p53-independent cell death of mature leukocytes^29^. To define the role of mature leukocyte death in radiation-induced lymphomagenesis of shp53 mice, we treated shp53 and control mice with 1.8 Gy × 4 TBI and administered dexamethasone 30 min after exposure to the first and last dose of radiation. Compared with TBI alone, the combination of TBI with dexamethasone caused a significant decrease in total thymocytes in both control and shp53 mice 1 and 4 days after irradiation as well as a decrease in whole bone marrow (WBM) cells in shp53 mice 7 days after irradiation (Supplementary Fig. 4a,b). However, dexamethasone treatment during TBI did not markedly change the number of LSK cells in the bone marrow (Supplementary Fig. 4a,b). When mice were followed for the development of cancer, dexamethasone treatment during TBI did not sensitize shp53 mice to radiation-induced lymphoma (1 out of 23 mice; Supplementary Fig. 4c). These results indicate that temporary knockdown of p53 during TBI suppresses lymphomagenesis via mechanisms that are independent of preventing the death of mature leukocytes.

### Blocking p53 during TBI improves HSPC fitness

**Figure 5:**
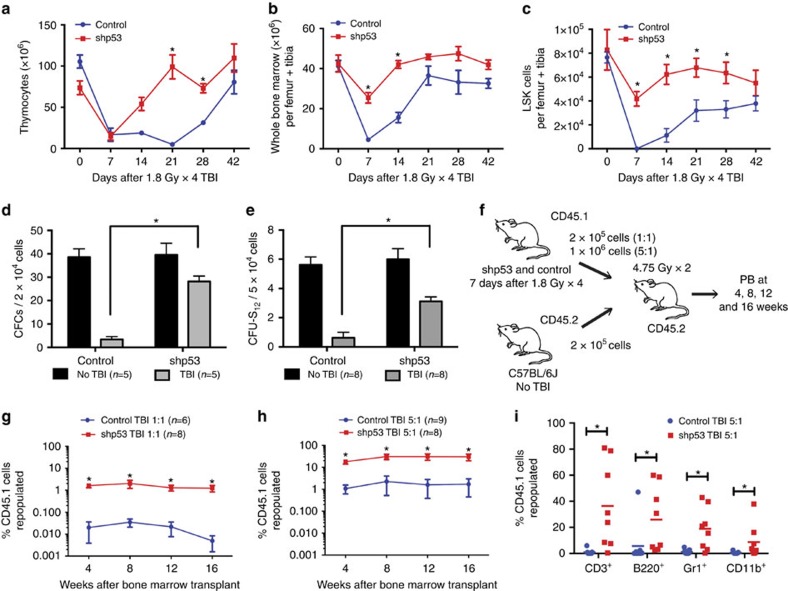
Temporary knockdown of p53 during TBI improves function of haematopoietic stem/progenitor cells. Quantification of thymocytes (**a**), WBM cells (**b**) and LSK cells in the bone marrow (**c**) at various time points after 1.8 Gy × 4 TBI (*n*=3–6 mice per group). *P<0.05 by two-way analysis of variance (ANOVA) with Bonferroni post hoc test. Data are presented as mean±s.e.m. (**d**,**e**) The number of colony-forming cells and the number of colony-forming unit-spleen on day 12 per 2 × 10^4^ and 5 × 10^4^ WBM cells, respectively. WBM cells were harvested from control and shp53 mice 7 days after 1.8 Gy × 4 TBI or no TBI. *P<0.05 by two-way ANOVA. Data are presented as mean±s.e.m. (**f**) Schematic representation of the competitive repopulation assay. 2 × 10^5^ or 1 × 10^6^ WBM cells from *CMV-rtTA; TRE-p53.1224* mice (shp53) and littermate controls (control) on a CD45.1 background 7 days after 1.8 Gy × 4 TBI were mixed with 2 × 10^5^ WBM cells from unirradiated C57BL/6 J (CD45.2) mice and transplanted into lethally irradiated C57BL/6 J (CD45.2) recipients. The chimerism of CD45.1/2 in the PB was analysed 4, 8, 12 and 16 weeks after BMT. (**g**,**h**) The percentage of PB cells that were repopulated by CD45.1 donors 4–16 week after BMT. *P<0.05 by two-way ANOVA with Bonferroni *post hoc* test. Data are presented as mean±s.e.m. (**i**) Multi-lineage haematopoietic reconstitution by CD45.1 donors 16 weeks after BMT. *P<0.05 by Student’s *t*-test. Data are presented as mean.

Over 60 years ago, Kaplan and Brown^31^ first described non-targeted effects of radiation on thymic lymphoma development and demonstrated that protecting the bone marrow from radiation is sufficient to prevent the formation of thymic lymphoma^32^,^33^,^34^. These observations prompted us to evaluate the impact of temporarily blocking p53 during TBI on the function of HSPCs in the bone marrow. In the absence of radiation, knocking down p53 for 10 days did not change the frequency and quiescence of LSK and LSKCD48^−^CD150^+^ (LSK-SLAM) cells^35^ (Supplementary Fig. 5a–c). In addition, competitive repopulation assays showed that temporary knockdown of p53 in the absence of radiation did not significantly alter haematopoietic reconstitution of donor CD45.1^+^ cells in peripheral blood (PB) 4–16 weeks after bone marrow transplant (BMT; Supplementary Fig. 5d–f). However, temporarily blocking p53 during 1.8 Gy × 4 TBI significantly improved regeneration of haematopoietic cells in the thymus and in the bone marrow (Fig. 5a,b), which correlated with a marked protection of LSK cells (Fig. 5c). It has been shown that cKit is downregulated in some HSPCs after radiation^36^ and therefore the use of LSK cells may underestimate the pool of surviving HSPCs. Thus, we performed additional experiments to quantify CD45^+^EPCR^hi^CD48^−^CD150^+^ (EPCR-SLAM) cells^37^ because recent studies suggested that HSPCs maintain expression of EPCR and CD150 after radiation injury^36^,^38^. Flow cytometry analysis of EPCR-SLAM cells 6 days after TBI (Supplementary Fig. 6a) showed that shp53 mice had a significantly higher number of EPCR-SLAM cells compared with control mice (Supplementary Fig. 6b). Moreover, temporarily blocking p53 during TBI maintained the quiescence of EPCR-SLAM cells (Supplementary Fig. 6c,d), which we hypothesized would improve the fitness of irradiated HSPCs. Indeed, *in vitro* colony formation assays (Fig. 5d) and *in vivo* spleen colony formation assays (Fig. 5e) showed that temporary knockdown of p53 during TBI significantly increased the frequency of functional haematopoietic progenitors. Finally, we performed competitive repopulation assays to assess long-term engraftment of HSPCs that survived irradiation (Fig. 5f). Competitive repopulation assays using either a 1:1 or a 5:1 ratio of irradiated (CD45.1) to unirradiated (CD45.2) WBM showed that temporary knockdown of p53 during TBI significantly improved long-term haematopoietic reconstitution of irradiated HSPCs (Fig. 5g,h) in both lymphoid and myeloid lineages (Fig. 5i). Taken together, these data demonstrate that temporarily blocking p53 during TBI significantly facilitates the regeneration of haematopoietic cells by improving the fitness of HSPCs in the bone marrow.

### Improved HSPC fitness after TBI reduces lymphomagenesis

**Figure 6:**
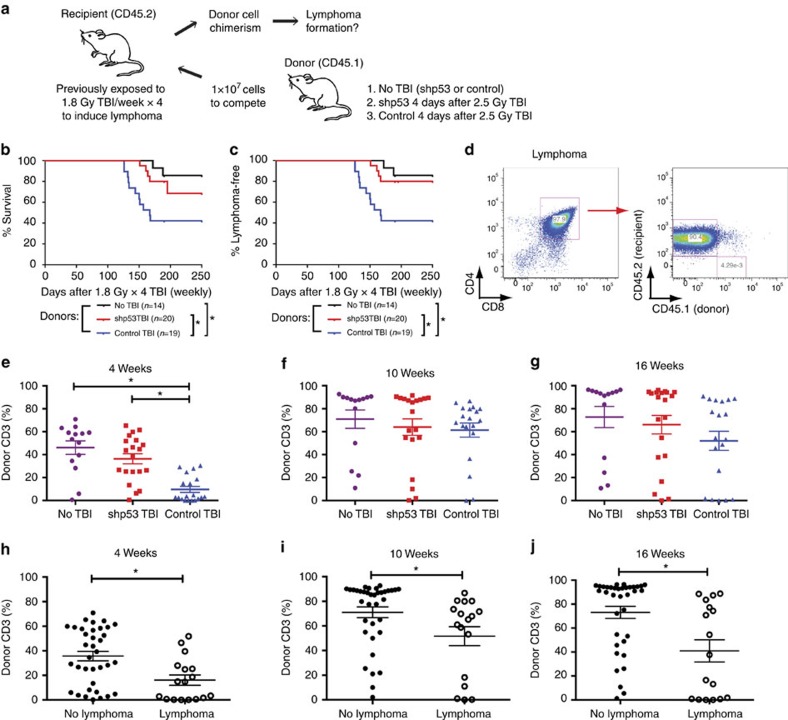
p53 in bone marrow cells responds to TBI to promote lymphoma development in the thymus. (**a**) Design of the BMT experiments that test the ability of donor bone marrow cells to suppress radiation-induced thymic lymphoma in recipient mice. C57BL/6 J (CD45.2) recipients were exposed to 4 weekly fractions of 1.8 Gy TBI to induce thymic lymphoma. Twenty-four hours after the last dose of TBI, these recipient mice were transplanted with 1 × 10^7^ WBM cells from *CMV-rtTA; TRE-p53.1224* mice (shp53; CD45.1) or littermate controls (control; CD45.1) 4 days after 2.5 Gy TBI or no TBI. The chimerism of CD45.1/2 in PB was analysed 4, 8, 12 and 16 weeks after BMT. (**b**,**c**) Overall and lymphoma-free survival of irradiated recipient mice that received bone marrow cells from different donors. *P<0.05 by log-rank test. (**d**) Representative flow cytometry plots of lymphoma cells that developed in recipient mice. (**e**– **g**) The percentage of CD3^+^ T-cells in PB repopulated by CD45.1 donors 4–16 weeks after BMT. *P<0.05 by Student’s *t*-test. Data are presented as mean±s.e.m. (**h**–**j**) The percentage of CD3^+^ T-cells in PB repopulated by CD45.1 donors 4–16 weeks after BMT in recipient mice that did (lymphoma) or did not (no lymphoma) develop lymphoma. *P<0.05 by Student’s *t*-test. Data are presented as mean±s.e.m.

To search for a causal link between the improved fitness of irradiated HSPCs in the bone marrow and the suppression of radiation-induced lymphomagenesis in the thymus, we examined whether the formation of radiation-induced thymic lymphoma in C57BL/6 J mice (CD45.2) can be inhibited after irradiation by transplantation with bone marrow cells from various donors. Donor mice (CD45.1) included unirradiated shp53 or control mice (no TBI), shp53 mice 4 days after 2.5 Gy TBI (shp53 TBI) or control mice 4 days after 2.5 Gy TBI (control TBI; Fig. 6a). We chose 2.5 Gy TBI because it causes a strictly p53-dependent decrease in HSPC fitness^39^. Consistent with previous studies^33^,^34^, prior irradiation significantly impaired the ability of bone marrow cells from control mice to suppress radiation-induced lymphoma in recipient mice (*P*=0.007 by log-rank test, no TBI versus control TBI; Fig. 6b,c). Remarkably, temporarily blocking p53 during TBI significantly restored the ability of irradiated bone marrow to suppress radiation-induced lymphomas (*P*=0.008 by log-rank test, shp53 TBI versus control TBI; Fig. 6b,c). Lymphomas that developed in irradiated recipient mice were mostly CD4^+^CD8^+^ and were positive for CD45.2, indicating that they were initiated from thymocytes of the recipient mice (Fig. 6d). Examination of donor (CD45.1) chimerism in PB 4, 10 and 16 weeks after BMT (Fig. 6e–g and Supplementary Fig. 7) showed that bone marrow cells from irradiated control mice had a significant defect in repopulating CD3^+^ T-cells in PB 4 weeks after BMT compared with bone marrow cells from unirradiated and irradiated shp53 mice (Fig. 6e). Notably, recipient mice that developed lymphomas had significantly lower donor (CD45.1) chimerism in CD3^+^ T-cells compared with mice that did not develop lymphoma (Fig. 6h–j). Together, these results demonstrate that the acute p53 response to radiation in bone marrow cells regulates the development of radiation-induced thymic lymphoma via a non-cell-autonomous mechanism.

## Discussion

**Figure 7:**
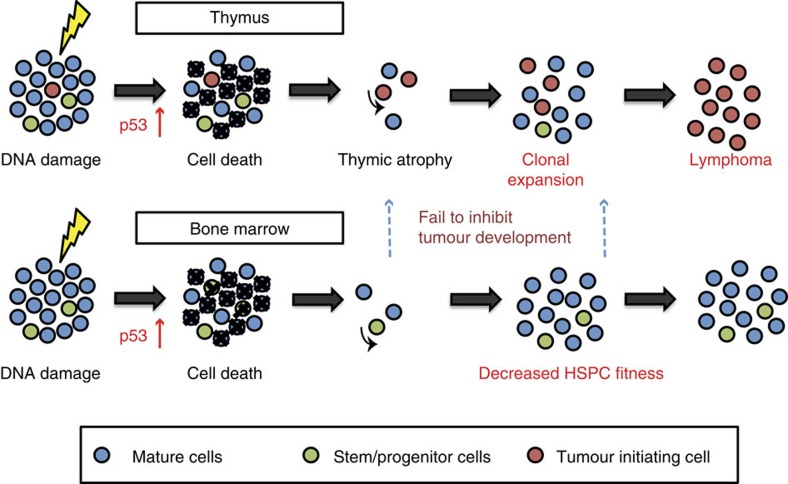
Proposed mechanism by which acute DNA damage activates p53 in the bone marrow to promote radiation-induced thymic lymphoma. TBI activates p53 to cause extensive cell death in the thymus and in the bone marrow (Figs 1 and 5). After irradiation, the decrease in the number and function of HSPCs in the bone marrow (Fig. 5) creates a favourable environment in the thymus that promotes the expansion of tumour-initiating cells (Figs 3 and 4). The expansion of lymphoma-initiating cells can be suppressed by unirradiated bone marrow cells or irradiated bone marrow cells from mice in which p53 is knocked down (Fig. 6), suggesting that radiation activates p53 to impair the ability of HSPCs and their progeny to compete with tumour-initiating cells in the thymus, which promotes the development of lymphoma (Fig. 2).

Emerging evidence from mouse models harbouring various point mutations in p53 demonstrate that the p53 response to acute DNA damage is dispensable for p53-mediated tumour suppression^40^,^41^,^42^. Here, our results from mice in which p53 activity was temporarily blocked during irradiation reveal a new paradigm for the role of the p53 response to acute DNA damage in radiation-induced lymphomagenesis: in p53 WT mice, the acute DNA damage response activates p53 in the bone marrow to promote radiation-induced lymphoma in the thymus via a non-cell-autonomous mechanism (Fig. 7).

Recently, bone marrow-derived thymic progenitors have been shown to suppress the development of spontaneous lymphomas via cell competition^23^. Our results suggest that the p53 response to acute DNA damage promotes radiation-induced thymic lymphoma, at least in part, by decreasing the ability of cells from the bone marrow to compete with tumour-initiating cells in the thymus. By reducing cell competition from the bone marrow, thymoyctes with an oncogenic mutation are more likely to expand and develop into a lymphoma after radiation exposure (Fig. 7). These results support a model of carcinogenesis in which tumour-initiating cells with adaptive mutations undergo clonal expansion as a result of impaired fitness of neighbouring stem/progenitor cells^25^,^39^,^43^,^44^. Moreover, this model provides a mechanism for the contribution of non-targeted effects to radiation-induced carcinogenesis, which was first described over 60 years ago^31^,^45^.

Finally, our findings have clinical significance because they support a model of therapy-related haematological malignancy in which genotoxic cancer therapies kill bone marrow cells in a p53-dependent manner so that rare tumour-initiating cells that already harbour an adaptive mutation preferentially expand as a consequence of treatment^46^. These data suggest that pharmacological inhibitors of p53 may not only ameliorate acute toxicity from chemoradiation by blocking apoptosis, but may also decrease the risk of therapy-related cancer at least in the haematopoietic system.

## Methods

### Study design and statistics

To search for a biologically meaningful effect size of p53 knockdown, sample sizes for experiments for carcinogenesis (Figs 2 and 4 and Supplementary Fig. 2), BMT (Fig. 6), and the acute radiation syndrome (Fig. 1) were estimated before initiating the study based on previously published work^21^,^33^,^34^,^47^.

For all experiments, data are presented as mean or mean±s.e.m. and each data point represents one mouse. Where variance was heterogeneous, data were log-transformed before applying statistical tests. Student’s *t*-test (two-tailed) was performed to compare the means of two groups. Two-way analysis of variance was performed to examine the interaction between genotypes and radiation treatment. Fisher’s exact test was performed to analyse data from the TCRβ locus rearrangement experiment. For carcinogenesis and haematopoietic acute radiation syndrome studies, Kaplan–Meier analysis was performed followed by the log-rank test. Significance was assumed at *P*<0.05. GraphPad Prism 5 (GraphPad Software, Inc.) was used for the calculation of the statistics.

### Mouse strains

All animal procedures for this study were approved by the Institutional Animal Care and Use Committee (IACUC) at Duke University. All of the mouse strains used in this study have been described previously including TRE-1224 (TRE-p53.1224 A), CMV-rtTA, Actin-rtTA, Kras^LA1^ and p53^−/−^ mice^17^,^18^,^26^,^48^. C57BL/6 J (CD45.2) and B6.SJL-*Ptprc*^*a*^ *Pepc*^*b*^/BoyJ (CD45.1) mice were purchased from The Jackson Laboratory. Four to 8-weeks-old male and female mice were used in the study. Mice on a mixed genetic background were used for all experiments except for BMT experiments. To perform BMT experiments, CMV-rtTA; TRE-p53.1224 mice were backcrossed to C57BL/6 J mice (CD45.2) for five generations, to B6.SJL-*Ptprc*^*a*^ *Pepc*^*b*^/BoyJ mice (CD45.1) for at least two generations, and then maintained on a CD45.1 background because we observed that the TRE-p53.1224 allele co-segregates with the CD45.1 allele. For experiments conducted using mice on a mixed genetic background, age-matched littermate controls were utilized to minimize the effect of genetic background. Therefore, potential genetic modifiers of the response to radiation would be randomly distributed among the experimental and control groups.

### Dox treatment

For radiation studies, all mice were fed a Dox-containing diet (6,000 mg kg^−1^ Dox, Cat. No. TD.04580, Harlan Teklad) for 10 days before irradiation, and were maintained on the Dox-containing diet until they received the last dose of radiation. Immediately after the last dose of irradiation, all mice were switched to a regular (Dox-free) diet. For unirradiated control studies, all mice were fed the Dox-containing diet for the same period of time as the irradiated groups and then switched to a regular (Dox-free) diet.

### Radiation treatment

TBI was performed 50 cm from the radiation source with a dose rate of 200 cGy min^−1^ or 220 cGy min^−1^ with 320 kVp X-rays, using 12.5 mA and a filter consisting of 2.5 mm Al and 0.1 mm Cu (X-RAD 320 Biological Irradiator, Precision X-ray). The dose rate was measured with an ion chamber by members of the Radiation Safety Division at Duke University.

### Dexamethasone treatment

250 μg of dexamethasone sodium phosphate (Sigma-Aldrich) in PBS was intraperitoneally injected into each mouse 30 min after irradiation. Age-matched littermates in the same cage were randomly selected to receive either vehicle or dexamethasone.

### Histological analysis

Tissue specimens were fixed in 10% neutralized formalin overnight, preserved in 70% ethanol, and then embedded in paraffin. Tumour diagnosis on haematoxylin and eosin-stained sections was performed by two veterinary pathologists (Y.K. and L.B.) blinded to the genotype or treatment. Immunohistochemistry staining was performed on tissue sections using anti-mouse CD3 (Thermo Scientific, clone: SP7, 1–200 dilution)^49^ or anti-pErk antibodies (Cell Signaling Technology, 1–500 dilution)^50^. The percentage of the lung with disease, the percentage of lung tumours positive for pErK, and the grade of the lung tumours were assessed without knowledge of genotype by a single observer (E.J.M.) following protocols described previously^51^ with a slight modification. Lung tumours were graded as low-, intermediate- or high-grade. Low-grade tumours formed a solid tumor and displayed regular to slightly irregular nuclei. Intermediate-grade tumours had enlarged, pleomorphic nuclei with prominent nucleoli and nuclear moudling. High-grade tumours displayed all of the characteristics of intermediate-grade lung tumours in addition to aberrant mitoses, tumour giant cells and desmoplasia.

### Immunoblotting

Proteins were extracted from cells or tissues using RIPA lysis buffer (Sigma-Aldrich). The protein concentration was determined using a bicinchoninic acid (BCA) protein assay kit (Thermo Scientific). A total of 30 mg of total protein were loaded for electrophoresis into 4–20% sodium dodecyl sulfate polyacrylamide gels (Bio-Rad). Separated proteins were transferred to a polyvinylidene difluoride membrane (Bio-Rad). Membranes were blocked with 5% BSA in tris-buffered saline (TBS) with 0.1% Tween 20. Protein levels were detected using antibodies against cleaved Notch1 (Val1744; Cell signaling Technology #2421, 1–1,000 dilution)^52^, c-Myc (Cell signaling Technology #2272 S, 1–1,000 dilution)^53^, p19Arf (Novus Biologicals, clone: 5-C3-1, 1–500 dilution)^54^, p-p53 (S15; Cell signaling Technology #9284 S, 1–500 dilution)^55^ and actin (BD Biosciences, 1–5,000 dilution) followed by secondary horseradish-peroxidase conjugated antibodies (GE Healthcare Life Sciences, 1–5,000 dilution). Bands were visualized using enhanced chemiluminescence (ECL) Plus western blotting detection reagents (Thermo Scientific). Uncropped scans of western blots can be found in Supplementary Fig. 8.

### Quantitative reverse transcription-PCR

Total RNA was extracted from samples using TRIzol reagent (Life technologies) and reverse transcription was performed using the iScript cDNA Synthesis Kit (Bio-Rad). Quantitative reverse transcription-PCR (qRT–PCR) was performed to detect mRNA expression using Taqman Universal Mix II (Applied Biosystems) with Taqman probes (Applied Biosystems, Mm01731290_g1 for *p53*, Mm00432448_m1 for *Cdkn1a*, Mm00449846_m1 for *Phlda3*, Mm01281680_m1 for *Lmo2*, Mm01342805_m1 for *Hes1* and Mm99999915_g1 for *Gapdh*). *Gapdh* was used as an internal control to correct for the concentration of RNA in different samples. Expression of miR-30-based p53.1224 shRNA was detected using a Custom TaqMan MicroRNA Assay (Applied Biosystems). A reverse transcription primer and a TaqMan probe were designed to specifically detect p53.1224 siRNA^56^. *In vitro* reverse transcription was performed using TaqMan MicroRNA Reverse Transcription Kit (Applied Biosystems). SnoRNA 202 was used as an internal control to correct for the concentration of RNA in different samples. Each experiment was performed with three replicates from each sample, and the results were averaged.

### Detection of V–J recombination at the TCRβ locus

V–J recombination at the TCRβ locus was analysed by PCR on genomic DNA using a pool of all Vβ forward primers with either Jβ1.7 or Jβ2.7 reverse primers that were described previously^23^ except the primer used for Vβ15 was corrected to 5′-GCTGGAGTTACCCAGACACCCA-3′. Genomic DNA was extracted from thymocytes or lymphoma tissues using DNeasy Blood & Tissue Kit (Qiagen) and quantified using NanoDrop (Thermo Scientific). PCR was performed using Platinum Taq DNA Polymerase High Fidelity (Invitrogen). PCR cycling conditions were described previously^23^.

### Quantification of the recombined Kras^G12D^ allele

Genomic DNA was extracted from thymocytes or lymphomas using DNeasy Blood & Tissue Kit (Qiagen) and quantified using NanoDrop (Thermo Scientific). To assess the frequency of the recombined *Kras*^*G12D*^ allele in *Kras*^*LA1*^ mice^26^, we first performed PCR to amplify a 3.5 kb DNA fragment of exon 1 of the WT *Kras* allele and the recombined *Kras*^*LA1*^ allele, which after undergoing intrachromosomal recombination is either WT *Kras* or *Kras*^*G12D*^. The forward primer: 5′-TGTAAGGCCTGCTGAAAATGACT-3′ and reverse primer: 5′-GACTGCTCTCTTTCACCTCC-3′ were selected so that they did not amplify the unrecombined *Kras*^*LA1*^ allele. PCR cycling conditions: 94 °C for 15 s, 60 °C for 30 s and 68 °C for 4 min for 30 cycles. The 3.5 kb size of this single PCR amplicon was confirmed by electrophoresis. PCR was performed using Platinum Taq DNA Polymerase High Fidelity (Invitrogen). The 3.5 kb PCR amplicon was purified using QiAquick PCR Purification Kit (Qiagen) and then used as a template for the subsequent droplet digital PCR experiment. For droplet digital PCR, we used the Primer Express software (Applied Biosystems) to design VIC and fluorescein amidite (FAM) conjugated Taqman MGB probes to specifically detect the WT *Kras* allele and the *Kras*^*G12D*^ mutant allele, respectively. The sequences of primers and probes for these two assays were:

Forward primer: 5′-TGTAAGGCCTGCTGAAAATGACT-3′

Reverse primer: 5′-TGTATCGTCAAGGCGCTCTTG-3′

Kras ^WT^ probe: 5′-TTGGAGCTGGTGGC-3′

Kras^G12D^ probe: 5′-TTGGAGCTGATGGC-3′

Digital PCR was performed using the RainDrop Digital PCR System (RainDance Technologies) following procedures described previously^57^. Standard PCR cycling conditions for Taqman-probe based PCR assay were used: cycling at 95 °C for 15 s and 60 °C for 1 min for 40 cycles. Data were analysed using the RainDrop Analyst data analysis software (RainDance Technologies) without knowledge of the genotype or treatment by Chris Miller from RainDance Technologies.

### Analysing thymocytes by flow cytometry

Total thymocytes were isolated from the thymus in haematopoietic stem cell (HSC) buffer (Hank's Balanced Salt Solution with Ca2^+^ and Mg2^+^, 5% fetal bovine serum, 2 mM EDTA). Red blood cells (RBC) were lysed using ammonium-chloride-potassium (ACK) lysing buffer (Lonza). Total number of thymocytes was counted with a Coulter counter (Beckman Coulter). 1 × 10^6^ thymocytes were blocked with a rat anti-mouse CD16/32 antibody (BD Pharmingen) and stained with phycoerythrin (PE)-Cy5 conjugated anti-mouse CD4 (clone: GK1.5), PE-conjugated anti-mouse CD8 (clone: 53-6.7), allophycocyanin (APC) conjugated anti-mouse CD44 (clone: IM7), fluorescein isothiocyanate (FITC) conjugated CD25 (clone: PC61.5) and APC-eFluor780 conjugated anti-mouse cKit (clone: 2B8) antibodies (eBioscience). All antibodies were diluted 1:400. Dead cells were excluded by staining with Calcein Blue AM (Life technologies). Data were collected from 250,000 single cells by FACSCanto (BD Pharmingen) and analysed by FlowJo (Tree Star, Inc.) without knowledge of the genotype or treatment by a single observer (C.L.L.).

### Analysing HSPCs in the bone marrow

WBM cells were isolated from femurs and tibias by grinding the bones in HSC buffer. RBCs were lysed using ACK lysing buffer (Lonza). Total number of WBM cells was counted with a Coulter counter (Beckman Coulter). Three million WBM cells were blocked with a rat anti-mouse CD16/32 antibody (BD Pharmingen) and stained with PE-Cy5 conjugated anti-mouse CD3e (clone: 145-2C11), PE-Cy5 conjugated anti-mouse CD4 (clone: GK1.5), PE-Cy5 conjugated anti-mouse CD8 (clone: 53-6.7) PE-Cy5 conjugated anti-mouse B220 (clone: RA3-6B2), PE-Cy5 conjugated anti-mouse CD11b (clone: M1/70), PE-Cy5 conjugated anti-mouse Gr-1 (clone: RB6-8C5), PE-Cy5 conjugated anti-mouse Ter119 (Ly-76), PE-conjugated anti-mouse Sca1 (clone:D7), APC conjugated anti-mouse cKit (clone: 2B8), FITC conjugated anti-mouse CD48 (clone: HM48-1; eBioscience) and Brilliant Violet 421 conjugated anti-mouse CD150 (clone: TC15-12F12.2; BioLegend) antibodies. All antibodies were diluted 1:400. Dead cells were excluded by staining with 7AAD (BD Pharmingen). Data were collected from 1 million single cells by FACSCanto (BD Pharmingen) and analysed by FlowJo (Tree Star, Inc.) without knowledge of the genotype or treatment by a single observer (C.L.L.).

### Analysing the cell cycle of EPCR-SLAM cells

3 × 10^6^ RBC-lysed WBM cells were blocked with a rat anti-mouse CD16/32 antibody (BD Pharmingen) and stained with APC conjugated anti-mouse CD45 (clone: 30-F11; eBioscience), PE-conjugated anti-mouse CD201 (EPCR; clone: eBio1560; eBioscience), APC-eFluor780 conjugated anti-mouse CD48 (clone: HM48-1; eBioscience) and Brilliant Violet 421 conjugated anti-mouse CD150 (clone: TC15-12F12.2; BioLegend) antibodies. All antibodies were diluted 1:400. Stained cells were fixed in HSC buffer containing 2% paraformaldehyde overnight at 4 °C. Fixed WBM cells were permeabilized using HSC buffer containing 0.025% Triton X-100 followed by Cytofix/Cytoperm Fixation/Permeabilization Solution Kit (BD Pharmingen). Permeablized WBM cells were stained with a FITC conjugated anti-human Ki67 antibody (BD Pharmingen) and then resuspend in HSC buffer containing 7AAD (BD Pharmingen). Data were collected from 1 million single cells by FACSCanto (BD Pharmingen) and analysed by FlowJo (Tree Star, Inc.) without knowledge of the genotype or treatment by a single observer (C.L.L.).

### Quantification of cell apoptosis

WBM cells and thymocytes were isolated following the same procedure described above. Cleaved caspase-3 staining was performed using PE Active Caspase-3 Apoptosis Kit (BD Pharmingen) according to the manufacturer’s instructions. Data were collected from 30,000 single cells by FACSCanto (BD Pharmingen) and analysed by FlowJo (Tree Star, Inc.) without knowledge of the genotype or treatment by a single observer (C.L.L.).

### HSPC colony formation assays

For *in vitro* colony formation assays, RBC-depleted WBM cells were isolated and counted following the same procedure described above. WBM cells were plated in MethoCult GF 3434 (Stem cell technologies) and colonies were counted 7 days later. Experiments to assess colony-forming unit-spleen 12 was performed following the protocol described previously^58^. RBC-depleted WBM cells were isolated and counted following the same procedure described above. WBM cells were transplanted into C57BL/6 mice irradiated with 9.5 Gy TBI. Spleens were harvested 12 days after irradiation and preserved in Bouin’s solution for counting the colonies. Colonies were counted without knowledge of the genotype or treatment by a single observer (C.L.L.).

### Competitive repopulation assay

CD45.1 donor mice were placed on Dox and treated with TBI as in Fig. 1a. Seven days later, RBC-depleted WBM cells were isolated and counted following the same procedure described above. WBM from CD45.1 donors were mixed with WBM from C57BL/6 (CD45.2) competitors and then transplanted into 8-week-old C57BL/6 (CD45.2) mice 6 h after two fractions of 4.75 Gy TBI with an interval of 18 h. After BMT, recipient mice were treated with Septra water for 4 weeks to prevent infection. At 4, 8, 12 and 16 weeks after transplantation, PB was collected from anaesthetised mice by submandibular bleeding and mixed with PBS containing 5 mM EDTA. RBCs were separated from PB mononuclear cells (PB-MNCs) using Ficoll-Plaque (GE Healthcare Life Sciences) by centrifugation. PB-MNCs were blocked with a rat anti-mouse CD16/32 antibody (BD Pharmingen) and stained with a lymphoid cocktail including PE-Cy5 conjugated anti-mouse CD3e (clone: 145-2C11), PE-conjugated anti-mouse B220 (clone: RA3-6B2), FITC conjugated anti-mouse CD45.2 (clone: 104), and APC-eFluor780 conjugated anti-mouse CD45.1 (clone: A20) antibodies (eBioscience) or a myeloid cocktail including PE-Cy5 conjugated anti-mouse Gr1 (clone: RB6-8C5), PE-conjugated anti-mouse CD11b (clone: M1/70), FITC conjugated anti-mouse CD45.2 (clone: 104), and APC-eFluor780 conjugated anti-mouse CD45.1 (clone: A20) antibodies (eBioscience). All antibodies were diluted 1:400. Data were collected from at least 20,000 single cells by FACSCanto (BD Pharmingen) and analysed by FlowJo (Tree Star, Inc.) without knowledge of the genotype or treatment by a single observer (C.L.L.).

### Blocking radiation lymphomagenesis by BMT

The purpose of this experiment was to examine if radiation-induced lymphoma in recipient mice can be suppressed by transplanting bone marrow cells from different donors^33^,^34^. Donor mice were 4-week-old *CMV-rtTA; TRE-p53.1224* mice and their littermate controls on a CD45.1 background. These mice were fed a Dox-containing diet for 11 days. On day 11, mice were exposed to a single fraction of 2.5 Gy TBI or no irradiation. WBM cells were harvested from these donor mice 4 days after TBI following the protocol described above and used for the subsequent transplant experiment. Recipient mice, which were 6-week-old male C57BL/6 J (CD45.2) mice, were exposed to 4 weekly fractions of 1.8 Gy TBI to induce thymic lymphoma^21^. Six hours after the last dose of TBI, recipient mice were randomly selected by cage to receive 1 × 10^7^ WBM cells from donor mice with various genotypes and radiation treatment via intravenous injection. At 4, 10, 16 weeks after BMT, PB was collected following the protocol described above. PB-MNCs were blocked with a rat anti-mouse CD16/32 antibody (BD Pharmingen) and stained with FITC conjugated anti-mouse CD45.2 (clone: 104; eBioscience), APC-eFluor780 conjugated anti-mouse CD45.1 (clone: A20; ebioscience), PE-conjugated anti-mouse B220 (clone: RA3-6B2; eBioscience), PE-Cy5 conjugated anti-mouse CD11b (clone: M1/70; eBioscience), PE-Cy5 conjugated anti-mouse Gr-1 (clone: RB6-8C5; eBioscience ) and Brilliant Violet 421 conjugated anti-mouse CD3e antibodies (clone: 145-2C11; BioLegend). All antibodies were diluted 1:400. Data were collected from at least 20,000 single cells by FACSCanto (BD Pharmingen) and analysed by FlowJo (Tree Star, Inc.) without knowledge of the genotype or treatment by a single observer (C.L.L.).

## Additional information

**How to cite this article:** Lee, C.-L. *et al*. Acute DNA damage activates the tumour suppressor p53 to promote radiation-induced lymphoma. *Nat. Commun.* 6:8477 doi: 10.1038/ncomms9477 (2015).

## Supplementary Material

Supplementary Information Supplementary Figures 1-8
