# Supplementary material for: Acute DNA damage activates the tumour suppressor p53 to promote radiation-induced lymphoma
Source: Nat Commun. 2015 Sep 24;6:8477. doi: 10.1038/ncomms9477 (PMC4586051; doi:10.1038/ncomms9477)
Supplement: Supplementary Information — Supplementary Figures 1-8 [file ncomms9477-s1.pdf]

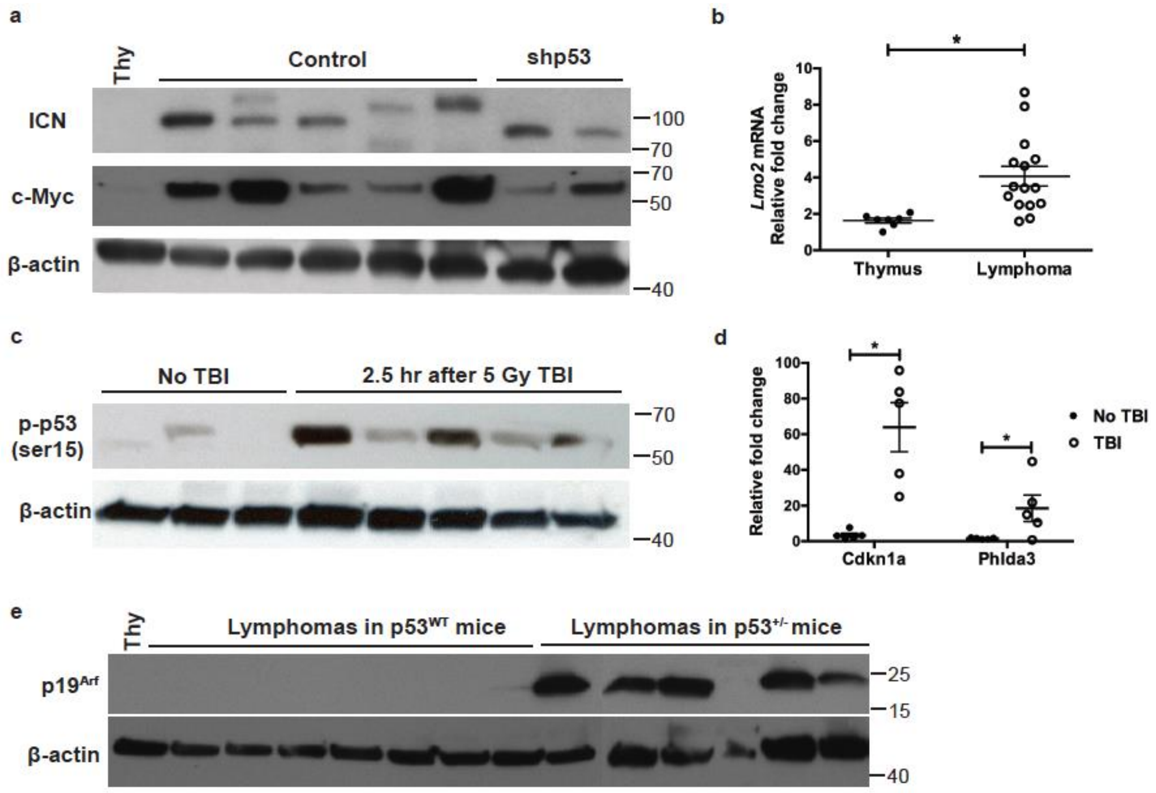

Supplementary Figure 1. Molecular characterization of radiation-induced lymphomas that developed in p53 wild-type mice. a, Examination of the intracellular domain of Notch (ICN) and c-Myc proteins in unirradiated thymus (Thy) and radiation-induced lymphomas that developed in either control or shp53 mice. **b,** Assessment of *Lmo2* mRNA in unirradiated thymi and radiation-induced lymphomas by quantitative RT-PCR. *P<0.05 by Students *t*-test. Data are presented as mean ± s.e.m. **c,d,** Examination of phosphorylation of p53 protein at Ser15 and mRNA expression of transcriptional targets of p53, *Cdkn1a* and *Phlda3,* in unirradiated lymphomas and lymphomas harvested from tumor-bearing mice 2.5 hours after 5 Gy TBI. *P<0.05 by Students *t*-test. Data are presented as mean ± s.e.m. **e***,* Assessment p19^{Arf} protein in unirradiated thymus (Thy) and in radiation-induced lymphomas developed in p53^{WT} or p53$^{+/-}$ mice. β-actin was used as a control for protein loading.

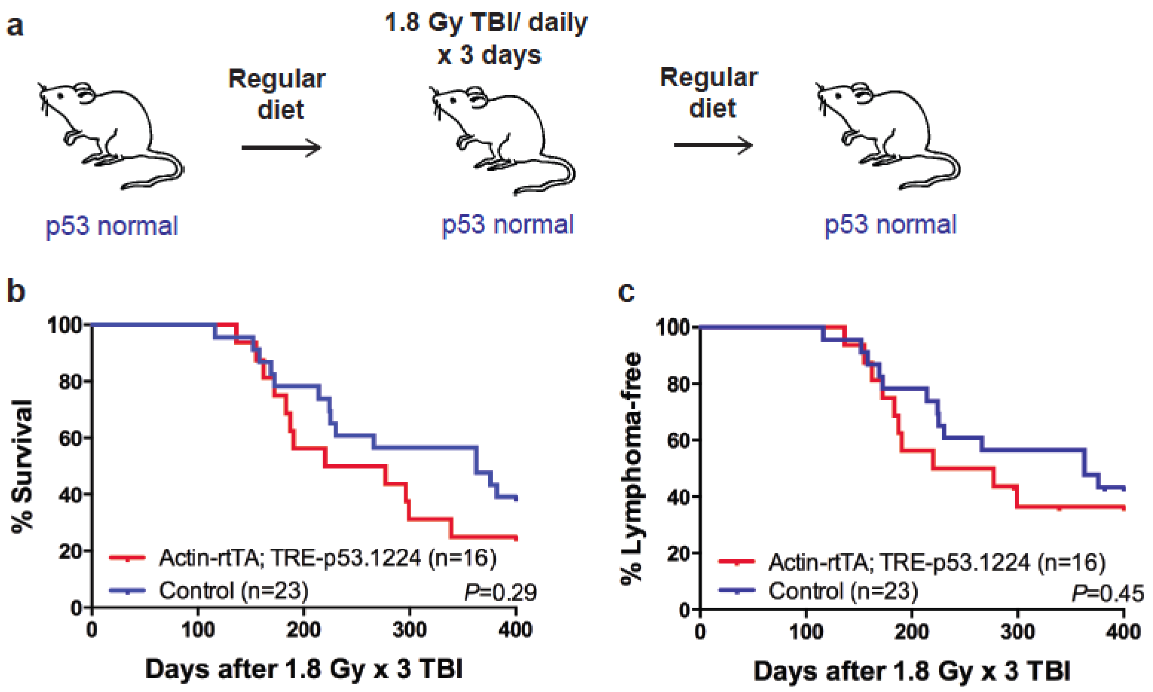

Supplementary Figure 2. In the absence of doxycycline treatment *Actin-rtTA; TRE-p53.1224* mice and littermate controls have similar susceptibility to radiation-induced lymphoma. a, *Actin-rtTA; TRE-p53.1224* mice and littermates containing either an Actin-rtTA or a TRE-p53.1224 allele (Control) were exposed to 3 daily fractions of 1.8 Gy TBI in the absence of Dox treatment. Three fractions of 1.8 Gy were selected for this experiment because in the absence of doxycycline 4 fractions of radiation caused the acute hematopoietic syndrome. **b,c,** Overall and lymphoma-free survival of mice following irradiation. *P*-value was calculated by log-rank test.

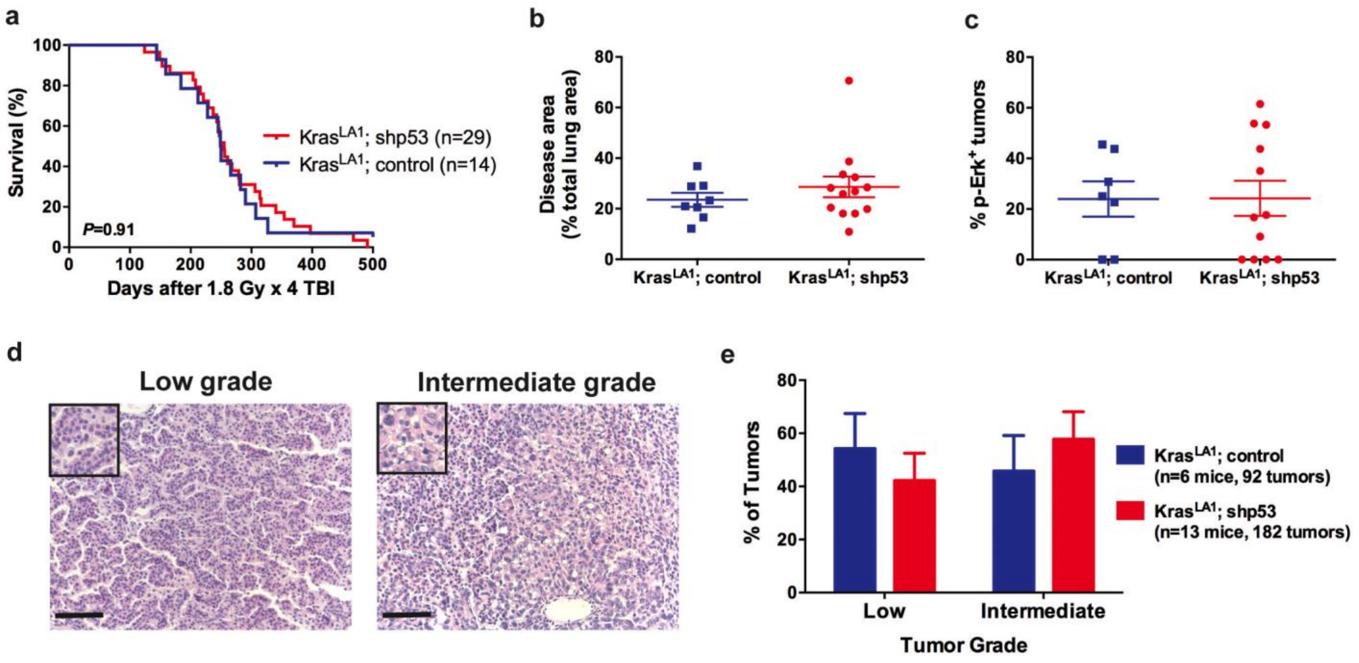

Supplementary Figure 3. Temporarily blocking p53 during total-body irradiation does not exacerbate lung tumor development in *Kras*^{LA1} mice. a, Overall survival of irradiated shp53 and control mice from Fig. 2b and c that did not develop lymphoma after TBI. *P*-value was calculated by log-rank test. **b,** The percentage of the lung (total lung area) containing lung tumors (disease area) in lymphoma-free irradiated shp53 and control mice. Data are presented as mean ± s.e.m. **c,** In each mouse, the percentage of lung tumors that contained >50% of the tumor area staining positive for phospho-p44/42 MAPK (p-Erk). **d,** Representative histology of low- and intermediate-grade lung tumors. Scale bar: 100 μm. **e,** The percentage of low- or intermediate-grade lung tumors in lymphoma-free irradiated shp53 and control mice. No high-grade tumors were observed. Data are presented as mean ± s.e.m.

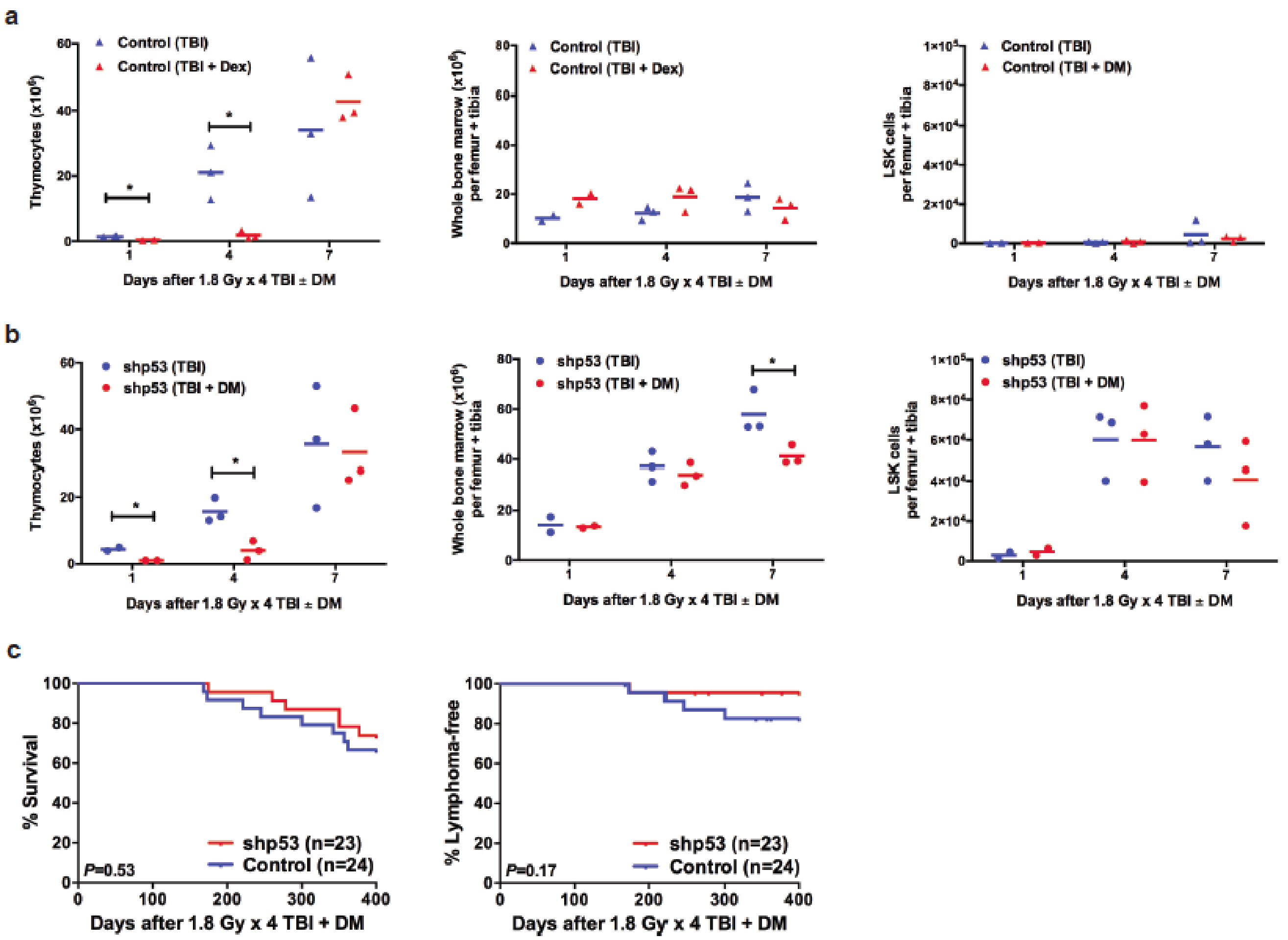

Supplementary Figure 4. Dexamethasone treatment during total-body irradiation causes p53-independent cell death of leukocytes, but does not sensitize mice to radiation-induced thymic lymphoma. *CMV-rtTA; TRE-p53.1224* (shp53) and littermates containing either an rtTA or a TRE- p53.1224 allele (Control) were fed a doxycycline (Dox)-containing diet for 14 days with exposure to 4 daily fractions of 1.8 Gy TBI starting on day 11. Mice were injected with vehicle (TBI) or dexamethasone (TBI + DM) 30 minutes after the first and last dose of TBI. **a,b,** The number of thymocytes, whole bone marrow cells and LSK cells in control and shp53 mice 1, 4 and 7 days after TBI ± DM treatment. ***P<0.05 by Student's *t* test. Data are presented as mean. **c,** Overall and lymphoma-free survival of shp53 and control mice following TBI + DM treatment. *P*-value was calculated by log-rank test.

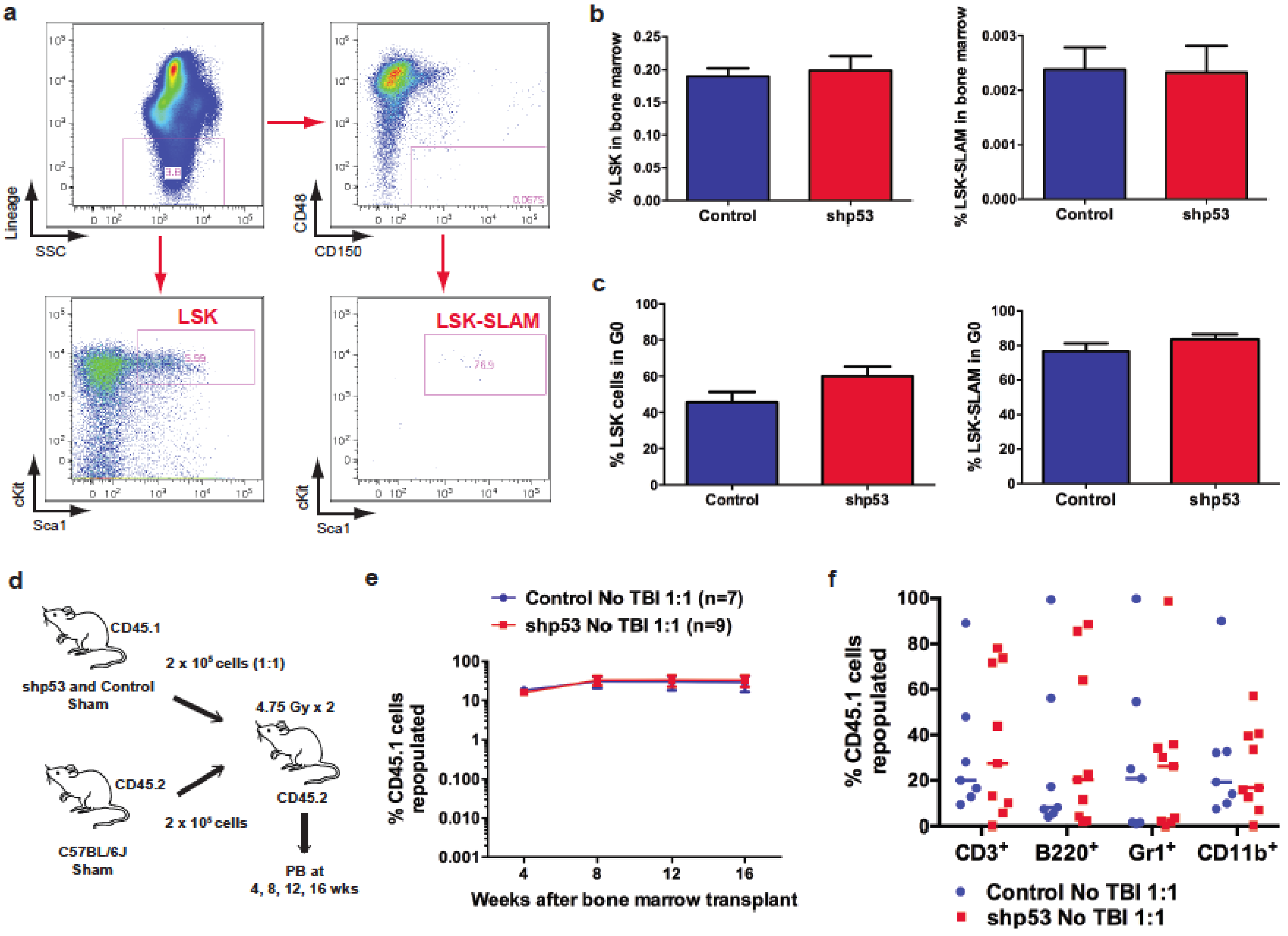

Supplementary Figure 5. Temporary knockdown of p53 for 10 days does not alter hematopoiesis under homeostasis. *CMV-rtTA; TRE-p53.1224* (shp53) and littermate controls with either a rtTA or a TRE-p53.1224 allele (Control) were fed a doxycycline (Dox)-containing diet for 10 days. **a,** Representative flow cytometry plots of Lineage$^-$ Sca1$^+$ cKit$^+$ (LSK) cells and LSKCD48$^-$CD150$^+$ (LSK-SLAM) cells in the bone marrow. **b,** The percentage of LSK and LSK-SLAM cells in unirradiated shp53 and control mice (*n* = 6 and n = 7 mice for control and shp53, respectively). Data are presented as mean ± s.e.m. **c,** The percentage of quiescent (G0) LSK and LSK-SLAM cells in unirradiated shp53 and control mice (*n* = 6 and n = 7 mice for control and shp53, respectively). Data are presented as mean ± s.e.m. **d,** Schematic representation of the competitive repopulation assay. 2 x 10^5 whole bone marrow cells from unirradiated shp53 and control mice on a CD45.1 background 7 days after Dox withdrawal were mixed with 2 x 10^5 whole bone marrow cells from unirradiated C57BL/6J (CD45.2) mice and transplanted into lethally irradiated C57BL/6J (CD45.2) recipients. The chimerism of CD45.1/2 in PB was analyzed 4, 8, 12 and 16 weeks after transplantation. **e,** The percentage of PB cells that were repopulated by CD45.1 donors 4 to 16 weeks after BMT. Data are presented as mean ± s.e.m. **f**, Hematopoietic reconstitution by CD45.1 donors 16 weeks after BMT in T-cells (CD3$^+$), B-cells (B220$^+$) and myeloid cells (CD11b$^+$ or Gr1$^+$). Data are presented as mean. Comparisons between control and shp53 mice were not significantly different by Student's *t* test (**b, c and f**) or by two-way ANOVA with Bonferroni post hoc test (**e**).

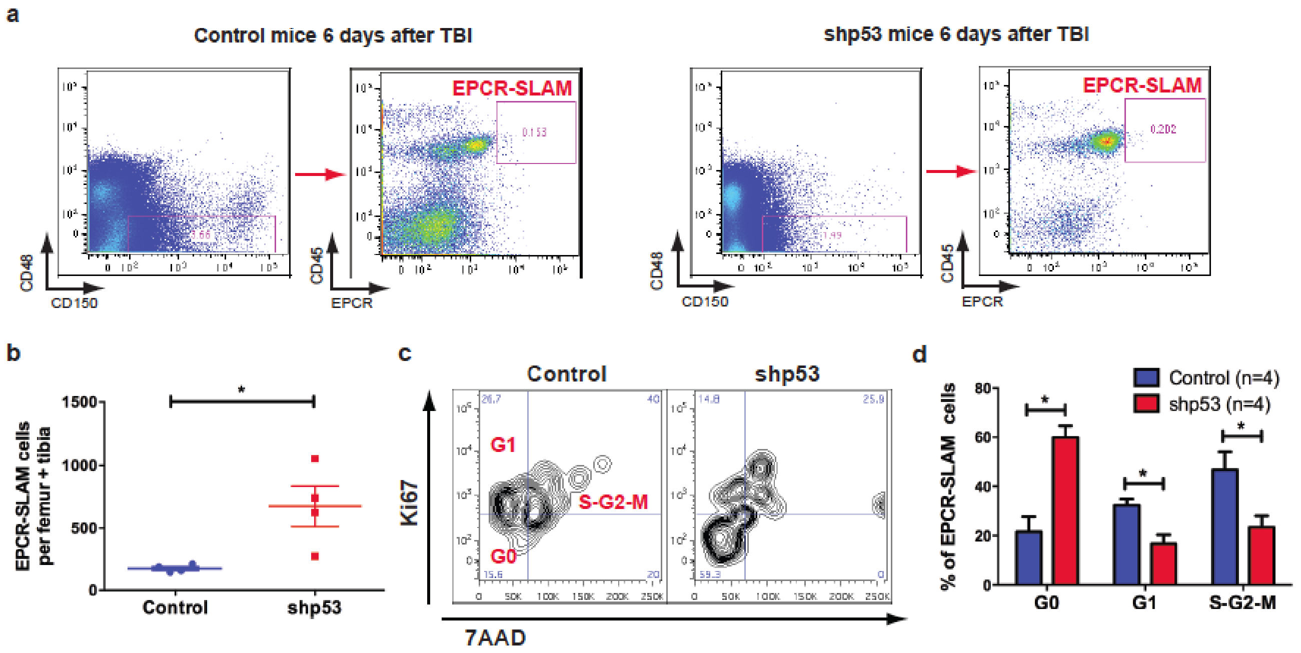

Supplementary Figure 6. Temporary knockdown of p53 during total-body irradiation maintains quiescence of EPCR-SLAM cells. a, Representative flow cytometry plots of $CD48^{-}CD150^{+}CD45^{+}EPCR^{hi}$ (EPCR-SLAM) cells in the bone marrow 6 days in control and shp53 mice after 1.8 Gy x 4 TBI. **b,** Quantification of EPCR-SLAM cells in the bone marrow 6 days after 1.8 Gy x 4 TBI. **P*<0.05 by Student's *t* test. Data are presented as mean ± s.e.m. **c,** Representative flow cytometry plots for cell cycle analysis. Cells at different stages of the cell cycle were distinguished by staining for Ki67 and with 7AAD. **d,** Quantification of EPCR-SLAM cells in control and shp53 mice at G0, G1 and S-G2-M phases of the cell cycle 6 days after 1.8 Gy x 4 TBI. **P*<0.05 by Student's *t* test. Data are presented as mean ± s.e.m.

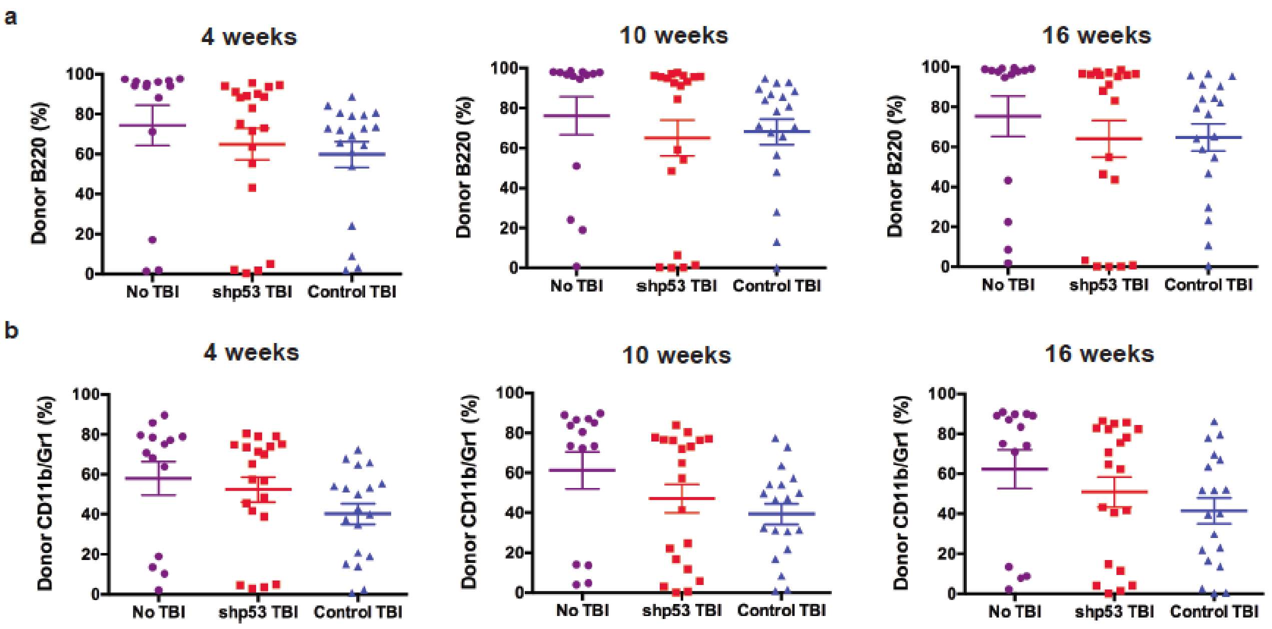

Supplementary Figure 7. B-cell and myeloid cell reconstitution from donor bone marrow cells in recipient mice that were irradiated with 4 weekly fractions of 1.8 Gy TBI. 1 x 10^7 whole bone marrow cells from *CMV-rtTA; TRE-p53.1224* mice (shp53) and littermate controls (Control) on a CD45.1 background 4 days after 2.5 Gy TBI or no TBI were transplanted into C57BL/6J (CD45.2) recipients 24 hours after 4 weekly fractions of 1.8 Gy TBI according to the protocol illustrated in Fig. 4a. The chimerism of CD45.1/2 in PB was analyzed 4, 8, 12 and 16 weeks after BMT. **a,b,** The percentage of $B220^+$ B-cells and $CD11b^+/Gr1^+$ myeloid cells in PB repopulated by CD45.1 donors 4 to 16 weeks after BMT. Comparisons between No TBI and control TBI mice or between shp53 TBI and control TBI mice were not significantly different by Student's *t* test. Data are presented as mean ± s.e.m.

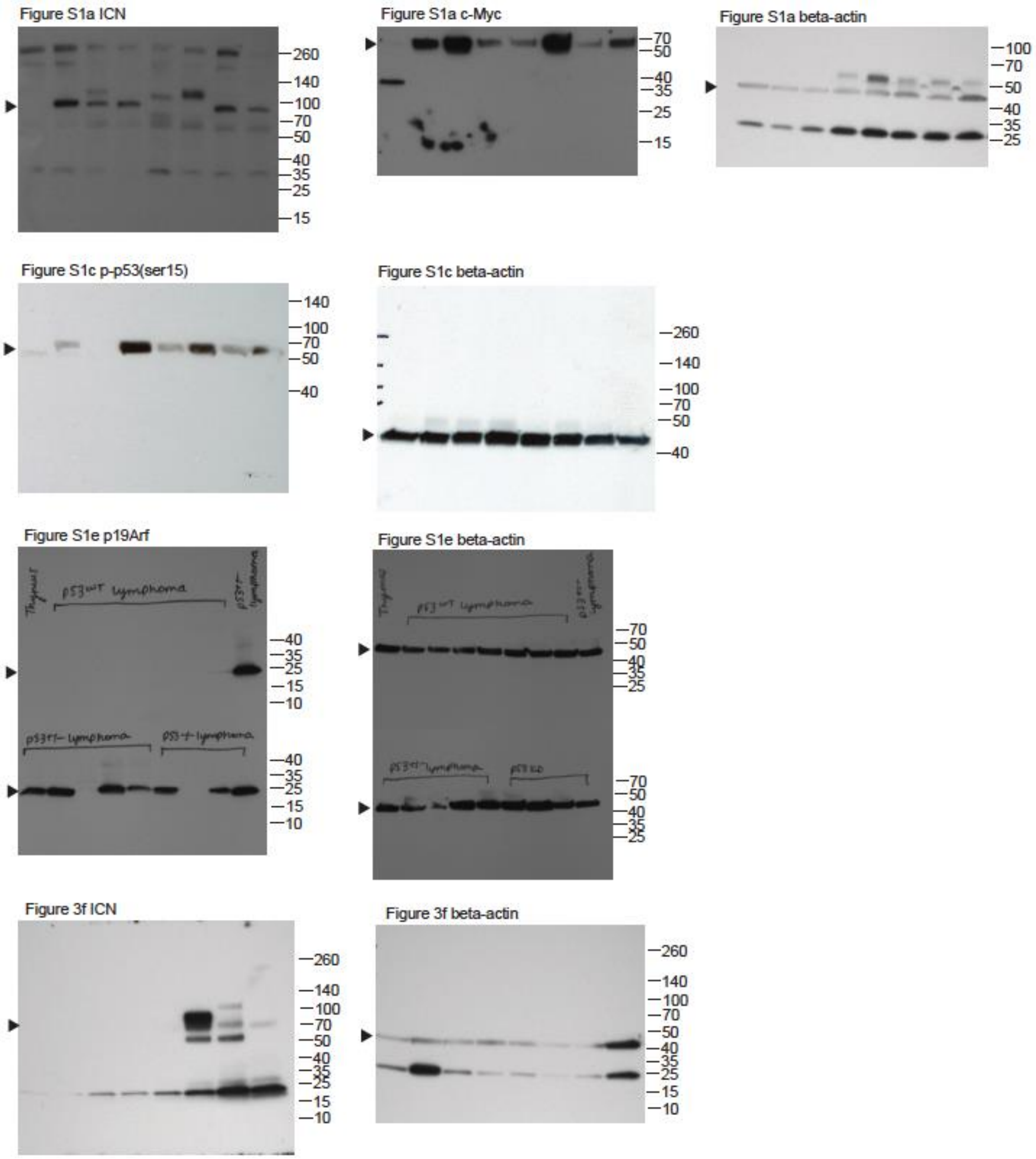

Supplementary Figure 8. Uncropped scans of Western blots.
